# Historical perspectives, challenges, and future directions of implantable brain-computer interfaces for sensorimotor applications

**DOI:** 10.1186/s42234-021-00076-6

**Published:** 2021-09-22

**Authors:** Santosh Chandrasekaran, Matthew Fifer, Stephan Bickel, Luke Osborn, Jose Herrero, Breanne Christie, Junqian Xu, Rory K. J. Murphy, Sandeep Singh, Matthew F. Glasser, Jennifer L. Collinger, Robert Gaunt, Ashesh D. Mehta, Andrew Schwartz, Chad E. Bouton

**Affiliations:** 1 Neural Bypass and Brain Computer Interface Laboratory, Feinstein Institutes for Medical Research, Northwell Health, Manhasset, NY USA; 2 Research and Exploratory Development Department, Johns Hopkins University Applied Physics Laboratory, Laurel, MD USA; 3 The Human Brain Mapping Laboratory, Feinstein Institutes for Medical Research, Northwell Health, Manhasset, NY USA; 4 Department of Neurosurgery, Barbara Zucker School of Medicine at Hofstra/Northwell, Manhasset, NY USA; 5 Department of Neurology, Donald and Barbara Zucker School of Medicine at Hofstra/Northwell, Manhasset, NY USA; 6 Departments of Radiology and Psychiatry, Baylor College of Medicine, Houston, TX USA; 7 Department of Neurosurgery, Barrow Neurological Institute, St. Joseph’s Hospital and Medical Center, Phoenix, AZ USA; 8 Good Shepherd Rehabilitation Hospital, Allentown, PA USA; 9 Departments of Radiology and Neuroscience, Washington University in St Louis, Saint Louis, MO USA; 10 Rehabilitation Neural Engineering Labs, University of Pittsburgh, Pittsburgh, PA USA; 11 Department of Physical Medicine and Rehabilitation, University of Pittsburgh, Pittsburgh, PA USA; 12 Department of Bioengineering, University of Pittsburgh, Pittsburgh, PA USA; 13 Center for the Neural Basis of Cognition, University of Pittsburgh, Pittsburgh, PA USA; 14 McGowan Institute of Regenerative Medicine, University of Pittsburgh, Pittsburgh, PA USA; 15 Department of Molecular Medicine, Donald and Barbara Zucker School of Medicine at Hofstra/Northwell, Manhasset, NY USA

## Abstract

Almost 100 years ago experiments involving electrically stimulating and recording from the brain and the body launched new discoveries and debates on how electricity, movement, and thoughts are related. Decades later the development of brain-computer interface technology began, which now targets a wide range of applications. Potential uses include augmentative communication for locked-in patients and restoring sensorimotor function in those who are battling disease or have suffered traumatic injury. Technical and surgical challenges still surround the development of brain-computer technology, however, before it can be widely deployed. In this review we explore these challenges, historical perspectives, and the remarkable achievements of clinical study participants who have bravely forged new paths for future beneficiaries.

## Introduction – historical perspective

Connecting electrically with the human brain and body dates back to the eighteenth century. While conducting experiments together, Luigi Galvani (1737–1798) and his wife, Lucia Galeazzi Galvani (1743–1788), discovered an electrical spark conducted to a nerve could activate an otherwise expired muscle (Whittaker 1989). They also experimented with special electrodes having dissimilar metals and hypothesized they were conducting ‘animal electricity’ from the animal itself to the muscle to cause the contractions they observed. Alessandro Volta (1745–1827), however, later contended it was not animal electricity at all but the dissimilar metals were the source of the electricity that caused the remarkable observations (Bresadola 1998). Regardless of the source, it became clear in the ensuing years that electricity not only helps govern motion but also the very thoughts that led to these marvelous discoveries. Years later, building on the work of the Galvanis, a physician and physiologist named Richard Caton (1842–1926) began to record electrical signals in the brains of rabbits and apes using a (aptly-named) galvanometer (Finger 2001). This was followed by psychiatrist Hans Berger (1873–1941), known as the father of the electroencephalogram (EEG), performing electrical brain stimulation and later the first brain recordings in humans (Nervenkrankheiten and 1929 n.d.). With these groundbreaking discoveries and experiments began our fascination with connecting the human brain to machines which continues still today.

Many decades later, the idea of interfacing with the brain to study movement and sensation became a rapidly growing trend in research. Early work in non-human primates involved indwelling electrodes placed in the motor area to study the associated electrical patterns during loaded and unloaded wrist movements (Evarts 1968). Later, the timing of firing patterns from individual neurons were used together to predict specific arm movements (Humphrey et al. 1970). The question of how force was encoded continued then further studied in experiments involving wrist movements against elastic loads and spike-triggered averages of muscle activity (Cheney and Fetz 1980). Later, the idea of directional tuning was born – where a motor neuron’s firing rate changes as a function of how much the direction of movement deviates from a ‘preferred’ direction (Georgopoulos et al. 1986; Kalaska et al. 1983). This led to the breakthrough work studying cortical representations of movements during drawing (Schwartz 1994) and 3D movement and robotic arm control in non-human primates (Taylor et al. 2002; Velliste et al. 2008). Following this, cortical control of muscle contractions in primates was demonstrated (Ethier et al. 2012; Moritz et al. 2008). Recordings of large groups (or populations) with arrays of electrodes were also performed in various areas which furthered the understanding of network behavior (Donoghue et al. 1998; Warland et al. 1997). Finally, stimulation-evoked sensations were studied to characterize percept thresholds in non-human primates (Romo et al. 1998) and later in rats (Butovas and Schwarz 2007).

With the strong scientific foundations laid by the turn of the millennia, a new chapter had begun that looked at the question of applying the knowledge gained to restoring independence for those impacted by disease or injury. One of the first clinical demonstrations of an implanted BCI/electrode (with one recording site) was in a person with ALS (Kennedy and Bakay 1998). The study participant was able to modulate her own neural signals in a binary fashion. The authors of this study envisioned that 1 day this type of BCI could control muscle stimulators and restore movement in paralyzed limbs. Before that could happen, subsequent studies used multi-electrode arrays to afford augmented communication and cursor control in persons living with movement impairment (Bouton 2009; Hochberg et al. 2006). This led to cortically-controlled robotic arms (Collinger et al. 2013; Hochberg et al. 2012) and ultimately restoration of thought-mediated movement in paralyzed humans as was previously imagined (Ajiboye et al. 2017; Bouton et al. 2016).

There have been many important technical developments, scientific questions raised, and important research efforts in the implantable BCI field for sensorimotor applications. These fall into areas that include: electrode design approaches, imaging, decoding methods, bridging damaged neural pathways, and sensory percepts and feedback. In this review, we will cover advances in each of these areas and discuss remaining challenges and future directions for this growing and exciting field.

### Electrode technologies and tradeoffs

**Fig. 1:**
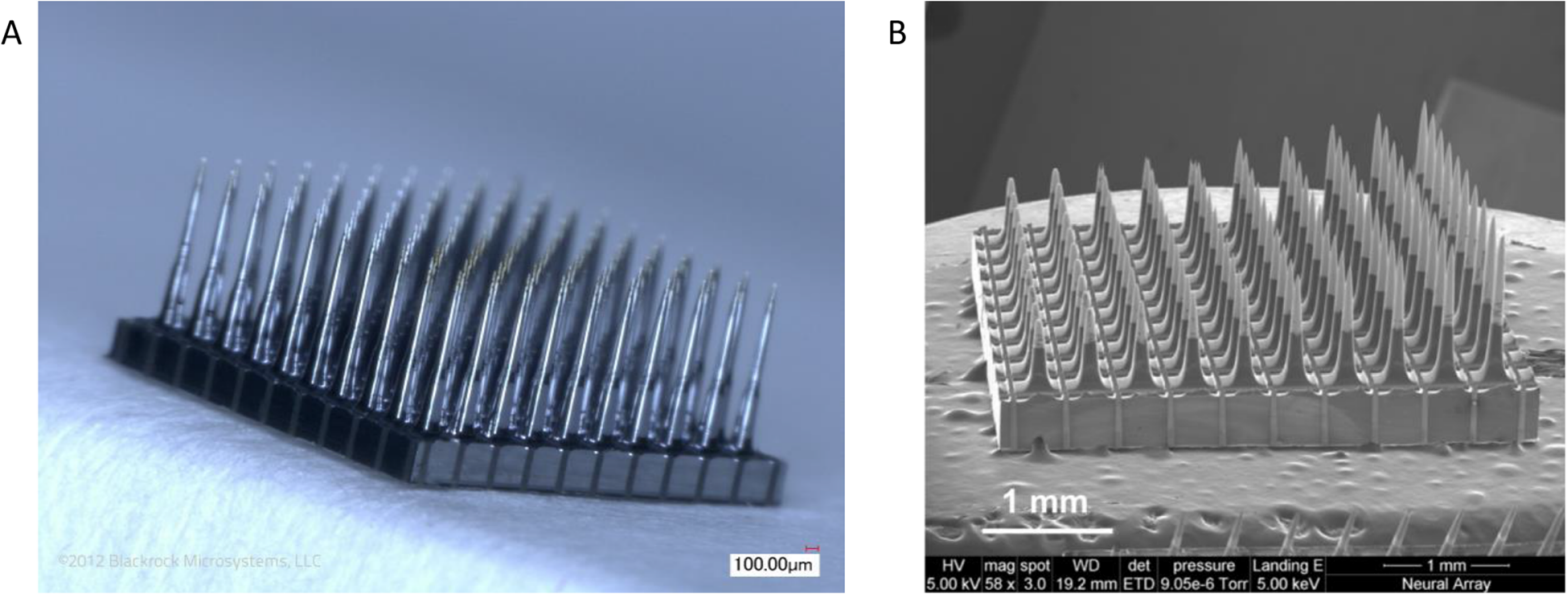
The Utah Array™. (**A**) Flat 96 electrode array fabricated by etching a solid piece of silicon followed by metallization, insulation, and wire bonding processes to create a final assembly. (**B**) Slanted array created for recording at electrical activity at various penetration depths. Photographs provided by Blackrock Microsystems, Inc.

Brain electrodes for recording or stimulation were typically made by hand in the early days of BCI research (and still are in some labs). Many new fabrication techniques, however, have since been developed and are employed for creating sophisticated devices. At the University of Utah, Richard Norman and his colleagues developed electrode arrays with many (often 96) electrodes by etching silicon to create ‘spikes’ and then subsequently metalizing them (Maynard et al. 1997) as shown in Fig. 1. Researchers at University of Michigan also created electrodes for brain recording and stimulation using a thin-film process that yielded flexible electrodes (Vetter et al. 2004) and another group created a so-called floating microelectrode array that allowed a variety of geometric layouts to be achieved (Musallam et al. 2007). The Utah, Michigan, and floating microelectrode arrays can be used for measuring single unit (neuron) activity, multi-unit activity, and local field potentials. Electrocorticography (ECoG) electrodes, which lay on the surface of the brain, are also commonly used in BCIs and can record electrical signal related to neuronal activity (Moran 2010; Wang et al. 2010, 2013). Standard ECoG arrays are not typically well-suited for measuring single unit/neuron activity but certain unique designs that are highly conformable to the brain’s surface have demonstrated this capability (Khodagholy et al. 2015). Furthermore, high electrode count/density devices have been developed and demonstrated in recent years. One particular implantable design has 455 electrodes with 51 active channels (Lopez et al. 2014). Also, a silicon probe design with over 5000 recording sites called Neuropixels has been developed to achieve high spatial and temporal resolution recordings of isolated neurons in cortex of small animals (Jun et al. 2017; Steinmetz et al. 2021). Lastly, Paradromics, Inc. has developed a 65,536 channel recording system which is comprised of a platinum-iridium microwire electrode array bonded to a CMOS (complementary metal oxide silicon) type voltage amplifier array recording from hundreds of neurons in rats and sheep (Sahasrabuddhe et al. 2020).

One important consideration with all of the types of implantable electrodes previously mentioned is the procedure required to install them. Microelectrodes, Utah arrays, ECoG grids, and other high count/density devices typically require a craniotomy which carries surgical risk and longer recovery time (Arya et al. 2013). The SEEG electrode, however, has been used extensively in recent years for mapping seizure origination acutely in epileptic patients and is now widely accepted (Whiting et al. 2020). Furthermore, the procedure (craniostomy) to insert SEEG electrodes is considered to be minimally-invasive and the adverse event rate is significantly lower than that with electrocorticography (ECoG) electrodes (Cardinale et al. 2013; Stricsek et al. 2018). SEEG electrodes therefore have high potential for future chronic BCI applications and recent studies show their performance may be comparable, and in some cases better than other ECoG electrodes for stimulation and decoding applications (Bouton et al. 2021; Chandrasekaran et al. 2021).

### Locating the target through imaging

Before implanting any type of BCI electrode, a critical first step is to image the brain and plan a suitable target site, such as the primary somatosensory and motor cortices. Though basic neuroanatomy is similar between people in and adjacent to the central sulcus, there are slight differences that make it difficult to precisely target small brain regions with microelectrodes. For example, representations of the thumb and pinky finger are separated by approximately 6 mm in the primary motor cortex (Dechent and Frahm 2003) and the size of a Utah array is approximately 4x4mm. Therefore, missing the target by even a few millimeters may result in missing neurons that are critical for BCI functionality.

Motor and somatosensory mapping is typically done both pre-operatively and intra-operatively. Structural magnetic resonance imaging (MRI) techniques can be used to identify basic neuroanatomical landmarks. Functional MRI (fMRI) scans can be aligned to structural MRI scans to further locate the target brain regions. For motor mapping, study participants attempt and imagine various movements of their hands or by observing hand movements during an fMRI (Bouton et al. 2016; Collinger et al. 2013; Hochberg et al. 2006; McMullen et al. 2021). For sensory mapping, study participants with somatosensory deficits can be asked to imagine tactile stimuli while in an fMRI (Fitzgibbon et al. 2012; Hodge et al. 1996). If individuals have intact motor and/or somatosensation, they can simply execute movements and receive tactile stimuli applied to the skin during an fMRI. In a recent case with invasive cortical microelectrode implantation, online functional mapping was used in conjunction with high-density electrocorticography (hd-ECoG) to localize finger areas in S1 (McMullen et al. 2021). Combined with traditional pre- and intraoperative targeting techniques, the researchers provided vibrotactile stimulation to finger regions of the study participant, who had intact somatosensation, during the operation. The hd-ECoG signals enabled targeted microelectrode array placement of neural regions that covered somatosensory finger representations. More recently, novel multi-modal MRI methods born out of the Human Connectome Project (Glasser et al. 2016) have been successfully used to identify cortical areas and somatotopic subregions of interest for implanting SEEG electrodes for both recording and stimulating in sensorimotor areas (Bouton et al. 2021; Chandrasekaran et al. 2021). Lastly, magneto-electroencephalogram (MEG) methods have also been used to image the brain in paralyzed participants who have metal implants that preclude use of MRI to identify suitable implantation areas (Flesher et al. 2016a, 2016b; Foldes et al. 2021; Goto et al. 2002).

### Decoding movement intentions

Being able to accurately decode movement intentions is crucial to enabling motor restoration or prosthetic limb control using BCI technology. A number of strategies and different machine learning algorithms including linear classifiers, regression models, support vector machines (SVMs), and deep neural networks have been used to decode neural signals recorded in the brain. Real-time neural decoding methods have been developed and demonstrated in humans with implanted Utah arrays (chronically) or ECoG electrodes (acutely). These decoding methods include a wide range of high-performance feature selection and machine learning techniques that allow high movement intention discrimination accuracy for gross and fine motor movements of the human hand in both paralyzed and able-bodied participants using Utah arrays (Bouton et al. 2016; Friedenberg et al. 2016a, 2016b, Friedenberg et al. 2017; Sharma et al. 2015, 2016a). Decoding of individual finger movement has also been demonstrated in ECoG recordings (Kubánek et al. 2009).

Despite having the advantage of being minimally-invasive, little work has been conducted to date on decoding signals recorded via SEEG electrodes for BCI applications. Basic two-dimensional cursor control was previously demonstrated via SEEG electrodes (Vadera et al. 2013), in which the user wiggled their contralateral hand or foot, to control the horizontal and vertical motion of a computer cursor respectively. Also, a BCI P300 Speller (single degree-of-freedom) was controlled through ECoG and SEEG electrodes implanted in and near the hippocampus (Krusienski and Shih 2011; Shih and Krusienski 2012). In another study, grasp force related events were recorded and classified using SEEG electrodes recording from sulcal areas in motor cortex and from sensory cortex (Murphy et al. 2016). Also, in a different study, three different hand gestures were decoded using SEEG signals with an accuracy of 78.70 ± 4.01% (Li et al. 2017a, 2017b). In a separate effort, SEEG electrodes placed in middle temporal regions led to typing of up to 14 characters/minute (Li et al. 2017a, 2017b). Furthermore, another group decoded SEEG recordings from the auditory cortex and produced intelligible waveforms with 45–75% accuracy levels depending on the algorithm used (Akbari et al. 2019).

Most recently, high accuracy decoding of both movement and sensory events was achieved in SEEG recordings using a temporal-correlation based (TCB) feature selection algorithm with deep learning methods (Bouton et al. 2021). It was shown that neural signals recorded from sulcal and subcortical areas contain useful information related to tactile stimuli and movement of individual fingers in able-bodied individuals and can be decoded accurately with long short-term memory (LSTM) type recurrent neural networks (RNNs) (Bouton et al. 2021). During actual finger movement and mechanical tactile stimuli (tapping) of the finger pads, phasic (transient) and phasic-tonic (transient-sustained) neural signals were identified, using temporal feature analysis, in all frequency bands analyzed across the 0 to 5000 Hz range. It was further shown that the TCB feature selection algorithm significantly improves decoding accuracy for both SVM and LSTM type algorithms when using SEEG (or HD ECoG) type electrode recordings in human participants. The mean decoding accuracy in SEEG recordings for finger movement tasks ranged from 86 to 92% (25% chance) and for tactile stimuli (tapping with Von Frey filament on finger pads), it ranged from 62 to 81% (25% chance) (Bouton et al. 2021)**.**

### Neural bypasses and bridges

Millions of people worldwide are suffering from sensory and motor impairments due to stroke, spinal cord injury, and other conditions, diminishing their quality-of-life (Armour et al. 2016). A BCI-based neural bypass or bridge, which re-routes signals around an injured portion of the nervous system, linking decoded signals to electrical stimulation of muscles or nerves, may restore movement and independence (Bouton 2018; Bouton 2020; Bouton et al. 2016; Friedenberg et al. 2016b; Sharma et al. 2016a). The first such bridge was demonstrated in primates using an implanted electrode array (Utah type) in the motor cortex which was linked to muscle stimulators (Moritz et al. 2008). In this study, monkeys were able to modulate their cortical activity and achieve bidirectional wrist movements. Graded grasping of multiple muscles was later demonstrated in primates as well (Ethier et al. 2012).

**Fig. 2:**
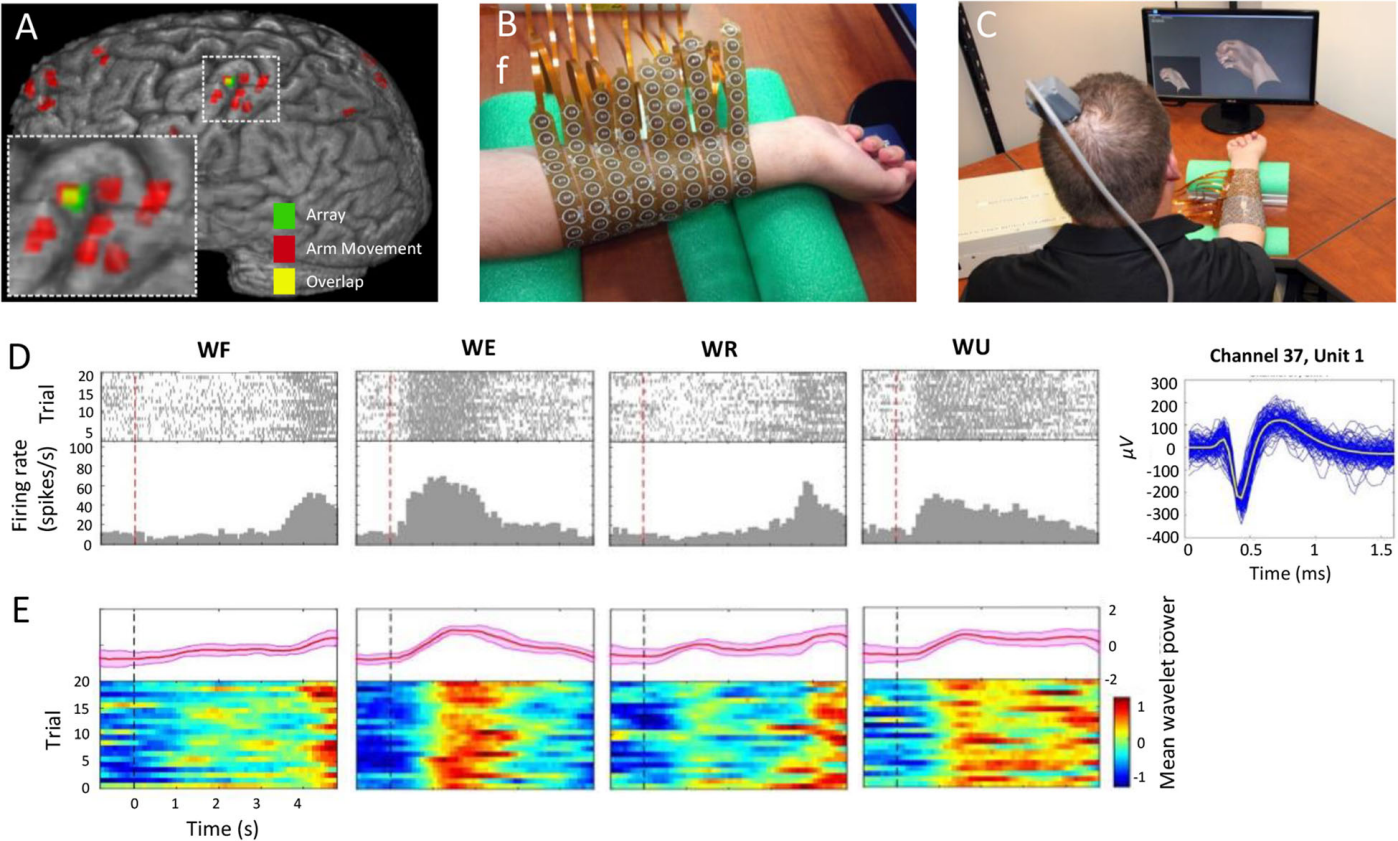
BCI system for movement restoration in a paralyzed human study participant. (**A**) Cortical implant location, (**B**) muscle stimulation sleeve, (**C**) experimental setup, and (**D**) raster plot of neural activity (channel 37, Unit 1) for imagined/attempted wrist movements (extension, flexion, and radial/ulnar deviations) and the unit temporal response, (**E**) mean wavelet power for all trials shown (bottom) and mean power (+/− 1 std. dev.) is shown in pink (top). Reprinted with permission from: *Bouton, Chad E.,* et al. *“Restoring cortical control of functional movement in a human with quadriplegia.” Nature 533.7602 (2016): 247–250*

The first human demonstration of restoring cortical control of volitional movement in paralysis using a neural bypass involved a BCI electrode array placed on the pre-central gyrus (primary motor cortex) of the brain (Bouton et al. 2016). This allowed decoding of hand and individual finger movements, and later, movements that were graded and even rhythmic (Friedenberg et al. 2017; Sharma et al. 2016b). As shown in Fig. 1, the neural bypass BCI system decoded (translated) neural activity into movement intentions which included specific finger movements, grasping movements, and as shown, wrist flexion and extension, along with ulnar and radial deviation (labeled as WF, WE, WR, and WU) Fig. 2.

**Fig. 3:**
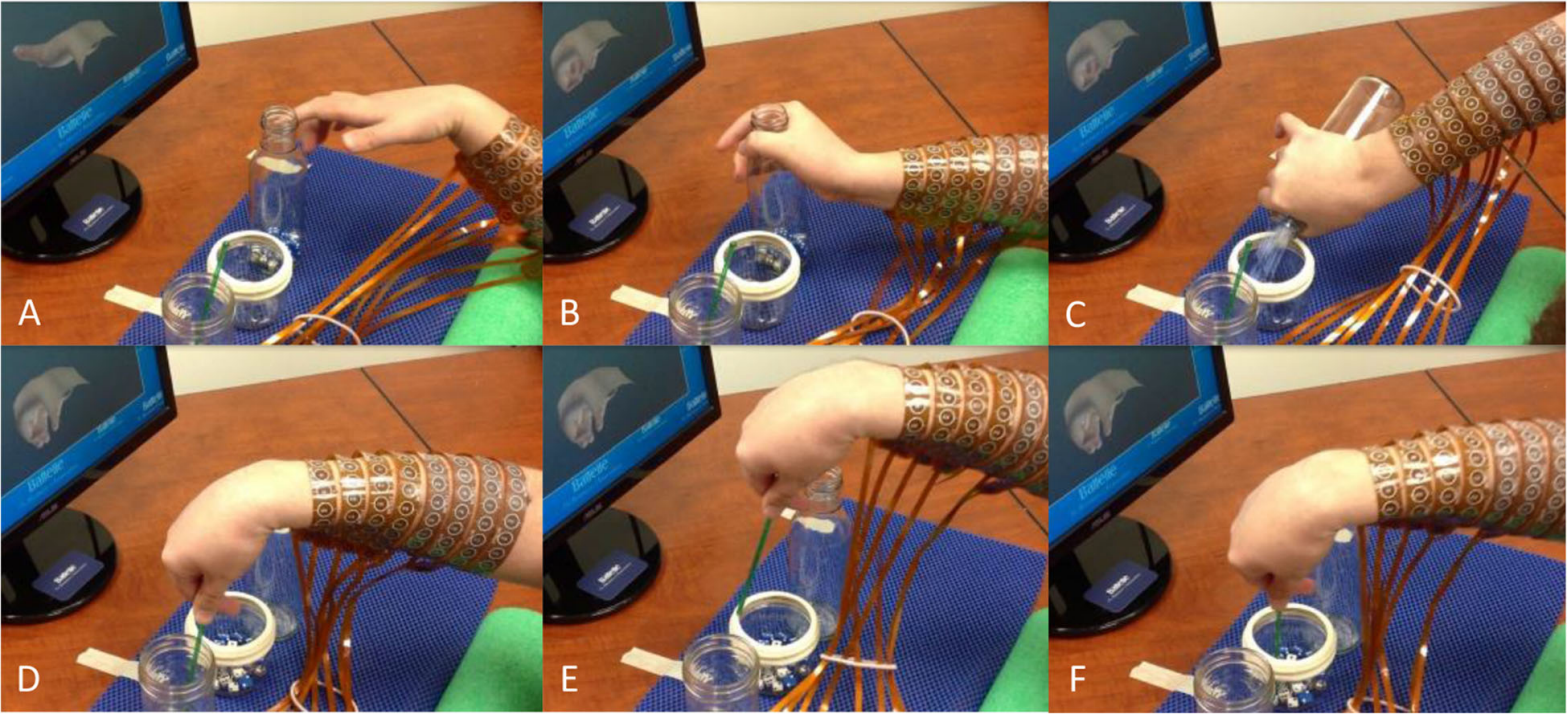
Functional movements achieved by a paralyzed study participant using an electronic neural bypass linking decoded brain activity to muscle activation in real-time. (**A**-**F**) Sequence of movements including opening of the hand, grasping a bottle with a cylindrical grasp, and stirring the contents with a pinch grasp. *Reprinted with permission from: Bouton, Chad E.,* et al. *“Restoring cortical control of functional movement in a human with quadriplegia.” Nature 533.7602 (2016): 247–250*

After the study participant became familiar with using the BCI-based neural bypass system and training of the neural decoding algorithms was completed, the participant was able to initiate and control various hand movements to manipulate different objects as shown in Fig. 3. The functional movements included: opening of the hand, grasping a bottle with a cylindrical grasp, and stirring the contents with a pinch grasp.

### Adding sensory feedback

Whether for improving prosthetic limb function in amputees or actual movements in people with paralysis, adding sensory feedback in a BCI system can significantly benefit sensorimotor functionality. Tactile percepts have been evoked in humans using intracortical microstimulation (ICMS) via microelectrode arrays (Fifer et al. 2020; Flesher et al. 2016a, 2016b; Salas et al. 2018) or cortical surface stimulation using electrocorticography (ECoG) grids (Hiremath et al. 2017; Kramer et al. 2021; Lee et al. 2018) in S1, specifically Brodmann’s area 1. This approach has been shown to improve prosthetic arm control performance, particularly in grasp confidence and transfer time (Flesher et al. 2021). Using biphasic ICMS pulses, reported tactile percepts include descriptions of pressure, squeezing, tapping, and vibration (Fifer et al. 2020; Flesher et al. 2016a, 2016b; Salas et al. 2018). Recently, researchers demonstrated that modulating the ICMS waveform being delivered to S1, specifically interpulse spacing, could lead to changes in the perceived tactile sensation, suggesting the ability to modulate perception through the stimulation waveform (Hughes and Gaunt 2021). Also, artificial proprioceptive feedback produced through intracortical microstimulation has enabled more accurate arm reaching in non-human primates (Dadarlat et al. 2015). Lastly, biomimetic stimulation approaches inspired by peripheral nerve recordings during mechanical stimuli have gained significant attention (Valle et al. 2018). To date, electrical stimulation in the somatosensory area of the brain does not produce completely natural percepts, but perhaps with further study and further development of biomimetic and other approaches, it may be possible in the future.

**Fig. 4:**
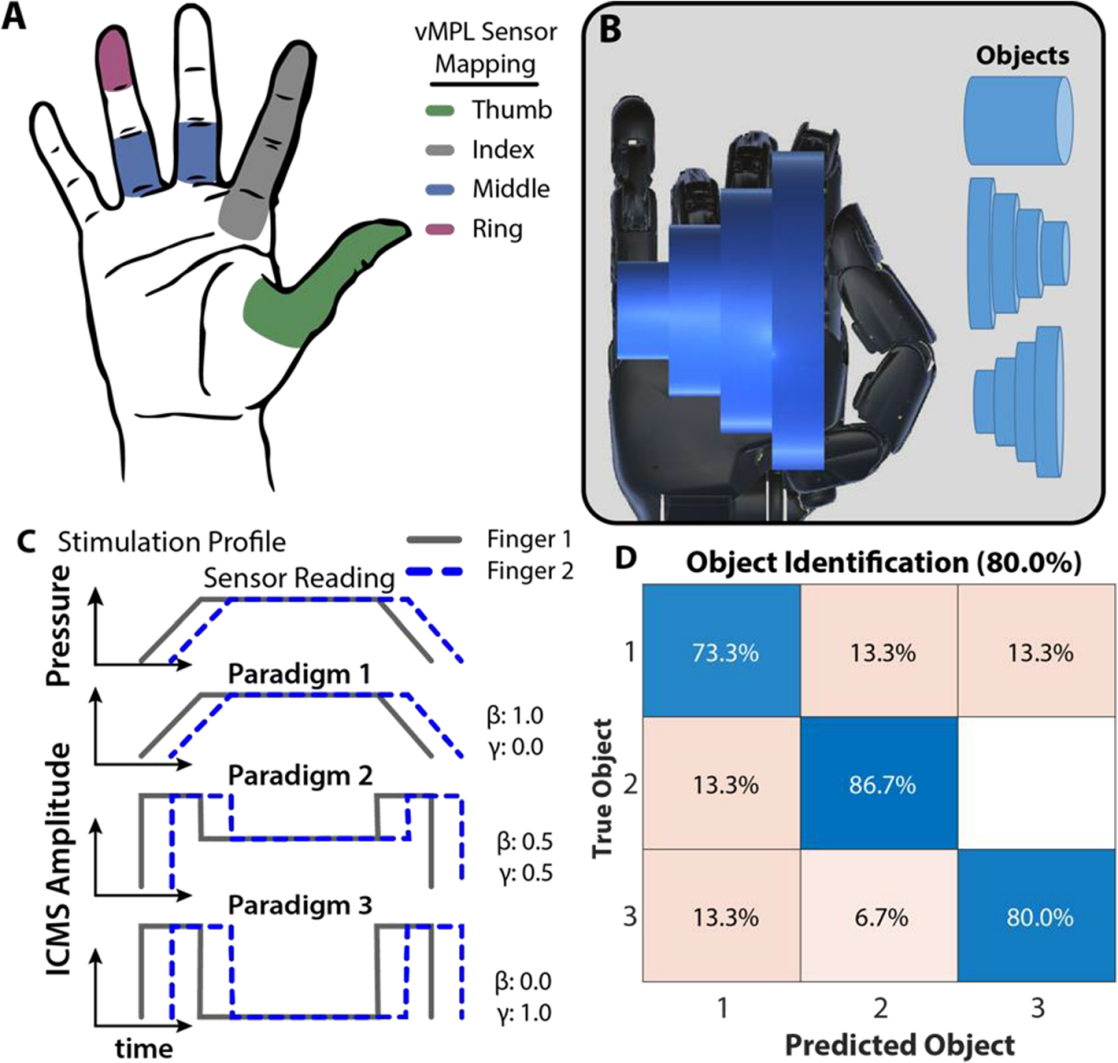
Object identification through stimulation-evoked tactile percepts. (**A**) Mapping of elicited tactile percepts and sensors from the virtual Modular Prosthetic Limb (vMPL) used for (**B**) grasping objects of varying shape. (**C**) ICMS amplitude was linearly modulated using different stimulation paradigms, each with a different weighting of sustained (β) and transient (훾) grip force. (**D**) Differences in the spatiotemporal tactile sensations restored through ICMS enabled the participant to identify the different objects. Image adapted and reproduced from (Osborn et al. 2021)

Recently, researchers showed the role of sensory feedback, through ICMS, to enable identification of different objects through touch while grasping with a virtual robotic limb. In a participant with microelectrode arrays in somatosensory and motor regions of the brain, amplitude modulated ICMS was delivered to provide spatiotemporal information during a virtual object grasping task through sensory feedback to the hand (Fig. 4) (Osborn et al. 2021). Touch sensors on a virtual robotic hand were mapped to projected fields on the participants hand and a linear weighting of sustained (β) and transient (훾) grip force was mapped to the ICMS amplitude to evaluate how different stimulation profiles enabled object identification through tactile sensations. The participant received sensory feedback on his intact hand based on sensory input on each of the virtual robotic hand’s fingers. With vision occluded, the participant used tactile input from the ICMS to identify between three different objects based on the unique spatiotemporal sensory perceptions generated by the ICMS. Identification performance reached accuracy up to 80%. The results demonstrate the functional use of sensory feedback through direct cortical stimulation for a relevant task, such as identifying different objects based on perceived shape. More broadly and relevant to the BCI field, the researchers showed that artifical sensory stimulation to brain regions can be perceived and incorporated by a human participant to accomplish a real-world task, thus helping set the stage for further investigation into how sensory percepts can be leveraged to enhance sensorimotor function.

**Fig. 5:**
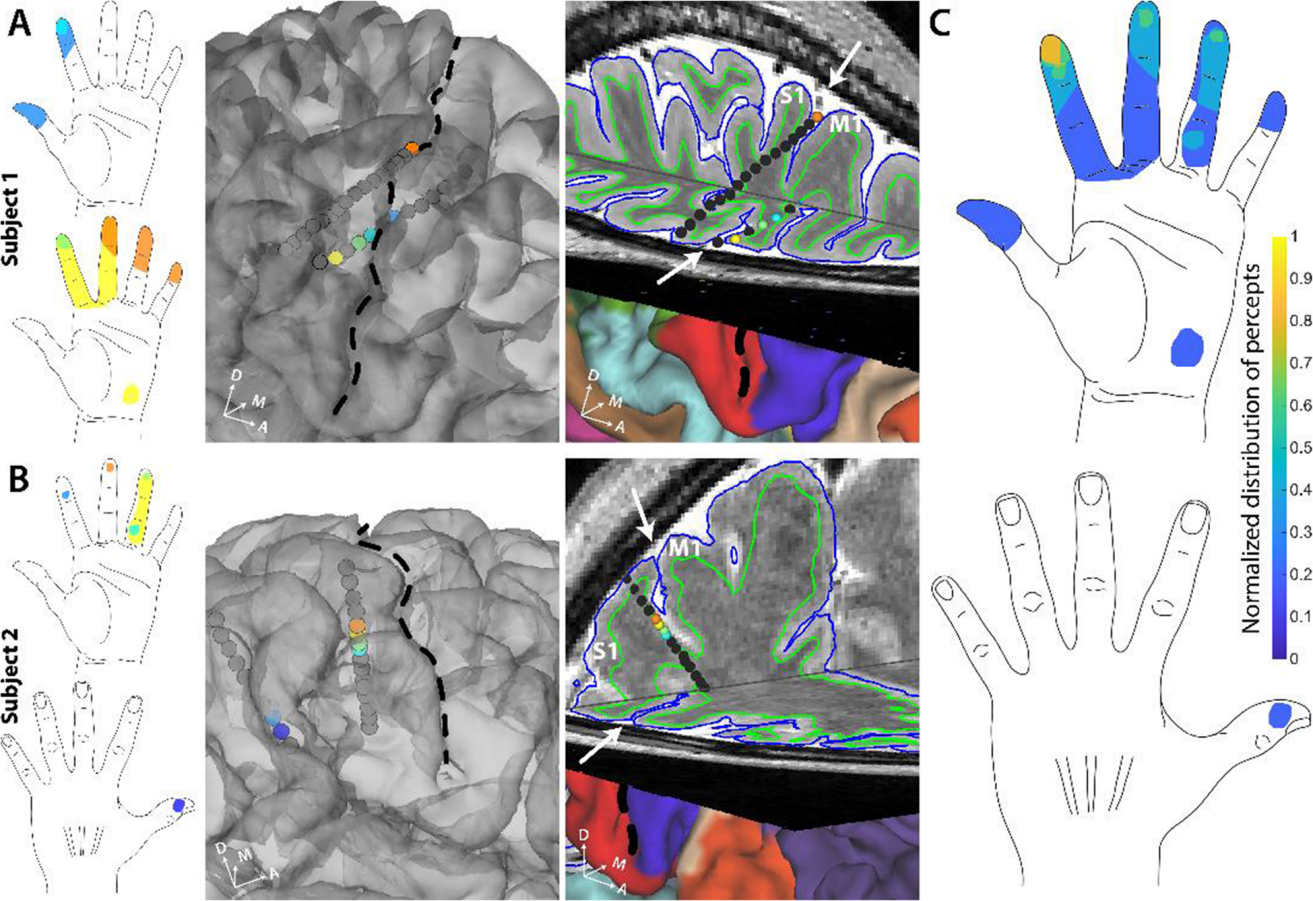
**A.** and **B.** Self-reported sensory percepts in the hand upon sulcal stimulation in S1. All the sensory percepts reported by participant 1 and 2 respectively upon SEEG-mediated sulcal stimulation. The color of each electrode matches the color of the corresponding percept evoked. The third panels show a 3D brain slice showing the same SEEG electrodes. Black dashed line and white arrows denote the central sulcus. **C.** Heatmap shows distribution of percepts evoked by S1 sulcal stimulation pooled from two participants. Number of percepts covering a region of the hand were normalized to the maximal number of percepts covering any area of the hand (*n* = 5). Image reproduced from (Chandrasekaran et al. 2021)

Achieving stimulation-evoked percepts at the fingertips, however, using intracortical electrodes has been difficult and requires extensive mapping to precisely locate the implantation site for the microelectrodes used (Fifer et al. 2020). In a first-of-its kind study, the representation of the hand, including the fingertips, in the sulcal regions of S1 was mapped using SEEG electrode based stimulation (Chandrasekaran et al. 2021). Upon electrical stimulation of these sulcal regions of S1, the participants reported tactile percepts that were localized to the contralateral arm and hand. Specifically, tactile percepts evoked by sulcal stimulation were highly focal and often located at or near the pads of the fingertips as shown in Fig. 5.

### Remaining challenges and future directions

Brain-computer interface technology is not currently ready for wide deployment. Fully implantable BCI electrode arrays and electronics can be associated with several implantation and design challenges. First, as discussed, there are surgical risks associated with any invasive devices, particularly those requiring a craniotomy for high-bandwidth devices (with high sampling rate and/or channel count) can require higher power levels which can lead to a shorter battery life. Also, most systems have been wired which cause concerns around impeding head movement, increased risk of infection, and noise artifacts caused by cable motion, but more recently wireless, high bandwidth percutaneous BCI devices have been developed to address these issues (Simeral et al. 2021). An immune response and signal degradation/instability can also occur over time in chronically implanted electrode arrays (Biran et al. 2007; Downey et al. 2018; Friedenberg et al. 2016b; Sharma et al. 2015).

Some researchers have argued an alternative to implanted BCIs is to use completely non-invasive technologies such as scalp-based electroencephalogram (EEG) or functional near-infrared spectroscopy (fNIRS) exclusively. EEG technology, for example, uses high sampling frequency (> 1000 Hz) but has been estimated to have a spatial resolution of only 6-8 cm (when 129 electrodes are used) (Ferree et al. 2001). Also, decoding performance in EEG for multiple hand/finger movements is typically lower than that of implantable BCIs such as ECoG and Utah arrays (Bouton et al. 2021; Liao et al. 2014; Shiman et al. 2017). Multichannel fNIRS, has also been proposed as an alternative non-invasive BCI, since it has an estimated spatial resolution of 2-3 cm (Pinti et al. 2020). However, fNIRS is limited to a 10 Hz sampling rate and is associated with latency of up to 2 s due to its dependence on slower hemodynamic phenomena (Frostig et al. 1990).

Recently, a novel approach of chronically deploying an electrode array via the vasculature of the brain by mounting them on a stent device has been pioneered (Oxley et al. 2016). With signal quality comparable to epidural and subdural recordings of neural activity (John et al. 2018), the ‘Stentrode’ device was recently shown to provide simple click activation (use with an eye tracking system for cursor control) to achieve typing in two patients with paralysis arising from ALS (Oxley et al. 2021). This approach provides the advantage of providing access to deeper structures of the brain, specifically the sulcal areas, owing to cerebral veins occurring in the sulcal folds.

Another avenue being explored is the use of high electrode count/density devices to record from significantly more neurons than possible with the devices previously mentioned. One particular design that was developed, for example, had 455 electrodes with 51 active channels (Lopez et al. 2014). Neuralink, co-founded by Elon Musk, is also developing a BCI with thousands of electrodes that are installed robotically (Pisarchik et al. 2019). Furthermore, Paradromics, Inc. recently developed a 65,536 channel recording system which is comprised of a platinum-iridium microwire electrode array bonded to a CMOS (complementary metal oxide silicon) type voltage amplifier array recording from hundreds of neurons in rats and sheep (Sahasrabuddhe et al. 2020). This technology needs to be miniaturized and packaging design challenges must be addressed before human deployment, but this an exciting approach that may open many new avenues for sensorimotor applications and even broader BCI applications.

## Conclusions

Our ability to interface with the brain has come a long way since the eighteenth century, but the fascination and growth of meaningful applications has been constant. With a wide range of conditions involving motor and/or sensory impairment, the need for BCI technology that can read, modulate, or even bypass compromised neurological pathways remains high. Many exciting new developments, methods, and technologies are underway, and on the horizon, paving the way to a bright future. The convergence of machine learning, electrode technology, and increased knowledge of the human nervous system will certainly give birth to more effective treatment options for patients - and perhaps even cures to conditions involving sensorimotor deficits one day.

## Data Availability

Not applicable.
